# On Treatment with Antituberculous Serum

**Published:** 1904-04-09

**Authors:** 


### ON TREATMENT WITH ANTITUBERCULOUS SERUM.

Dr. Alexander Marmorek,[1] speaking at St. George's Hospital, said that he had been working at this subject for a number of years, and was convinced that in the laboratory would be found the long sought for cure for tuberculosis. While not minimising the importance of hygienic measures, vaccination against tubercle, and the like, he emphasised the fact that what is wanted now is a cure for those already diseased.

The human organism is refractory to the tubercle bacillus: 20 per cent. of patients who already show signs of its invasion are enabled to recover by means of their natural immunity alone. Recoveries due to different curative methods amount to 20 or 22 per cent. Therefore, although no doubt the patient's condition is much ameliorated by sanatorium, and other such treatment, the proportion of cures is not much above the proportion of cases recovering by their natural immunity.

Tuberculin is not the primary cause of the pathogenic symptoms of tuberculosis. It is a reactive which causes the bacilli to secrete another and hitherto unknown toxin, and this toxin is the weapon with which the bacilli attack and undermine the organism. If an infinitesimal quantity of bacilli be injected beneath the skin of an animal, and after 30 minutes 1-80th of a drop of tuberculin be injected into the brain of both this animal and a control, a rise of temperature occurs in both, but is more marked in the inoculated animal. This genuine and precocious tuberculin reaction can only be explained by the fact that the injected tuberculin stimulated the bacilli which had been previously introduced under the skin to secrete a toxin causing fever. Thus the treatment by tuberculin aims at an active immunisation, and is unsuited to all forms of severe tuberculous intoxication and all cases where natural immunity is absent. Passive immunisation by introducing an antitoxin already produced would cure consumptives whether possessed of a natural immunity or not. An antitoxin prepared from the true toxin would have most effect upon those cases in which the intoxication is most severe, and when the symptoms are entirely due to the tuberculous process, and not to mixed infection or to the results of chronic inflammation. Dr. Marmorek believes that he has succeeded in producing the real tuberculous toxin in vitro,[2] and its corresponding antitoxin by methods of active animal immunisation. He treated at first only patients in the more advanced stages; his results encouraged him to use the serum in earlier and slighter cases. He found that a long series of injections caused very little local or general intolerance; that the younger the lesion the quicker were the healing effects of the serum to be expected.

The serum is directed against the intoxication in its primary effects. If such primary lesions alone are found a *restitutio ad integrum* can be accomplished. A woman of 33, with every classical symptom of acute miliary tuberculosis of both lungs, was treated by serum injection 27 days after the inception of the disease, all other treatment was excluded, and the case had been pronounced hopeless. After 720 cubic centimetres of serum had been introduced in 47 injections, absolute recovery was proved, and for months the sputum has been free from bacilli, and the signs in her chest have been normal. In the most acute form of tuberculosis, that of the meninges, the treatment has been commenced too late. Some cerebro-spinal fluid obtained by lumbar paracentesis should be used as the first injection in a tuberculin observation such as has just been described, and thus a diagnosis may be made quite early. The results in cases where the treatment has been commenced too late for cure have still been very encouraging; the condition of the patients was ameliorated at every point.

The serum is capable, Dr. Marmorek states, of curing pulmonary consumption by specific action. It has little or no effect upon too severe or chronic cases in which there are large cavities and irreparable lesions. The most recent portions of the infiltration are affected most favourably and very quickly resolve. The reconstruction of the tissue of the older and duller parts is more difficult and it is impossible to clear the portion of lung which is enveloped with pleuritic membrane and in which there is a large amount of fibrous tissue. Many injections may still be needed after symptoms of disease have disappeared to remove all bacillary trace from the sputum, and even cured patients continue for some time to expectorate several grammes of sputum daily.

Fever is reduced in all cases where the tubercle bacillus is the cause, but where there is a mixed infection it is sometimes useful to combine with the antituberculous an injection of antistreptococcus serum. During treatment there has been in the cases observed an almost entire absence of hæmoptysis. This may be accidental.

Surgical tuberculosis is rapidly and markedly affected by the injections, suppuration ceases, sinuses, intestinal ulcerations, and fistulæ heal, Pott's disease and hip-joint tuberculosis can be cured by the exclusive use of the serum. The dose employed is from 5 to 20 c.c., and after two or three daily injections an interval of two or three days is recommended, and after three or four series of injections they should be discontinued for a week or more. Some patients are intolerant to these doses. Occasionally "erythems" are noticed, but no serious consequences due to the injections have been noticed.

[1] Lancet, March 26, 1904. [2] Ibid., Dec. 12, 1903.

